# Socio-ecological factors of girl child marriage: a meta-synthesis of qualitative research

**DOI:** 10.1186/s12889-023-17626-z

**Published:** 2024-02-10

**Authors:** Asma Pourtaheri, Mehrsadat Mahdizadeh, Hadi Tehrani, Jamshid Jamali, Nooshin Peyman

**Affiliations:** 1 Student Research Committee, Mashhad University of Medical Sciences, Mashhad, Iran; 2 Department of Health Education and Health Promotion, School of Health, Mashhad University of Medical Sciences, Mashhad, Iran; 3 Department of Biostatistics, School of Health, Mashhad University of Medical Sciences, Mashhad, Iran; 4 Social Determinants of Health Research Center, Mashhad University of Medical Sciences, Mashhad, Iran

**Keywords:** Child marriage, Meta-synthesis, Qualitative research

## Abstract

**Background:**

Child marriage of girls is one example of human rights violations, and is increasingly recognized as a key obstacle to global public health. Given the importance of a comprehensive understanding of the motivations for child marriage, this study aimed to identify socio-ecological factors contributing to gills child marriage.

**Methods:**

A comprehensive search was conducted of all English-language studies measuring causes of child marriage between 2000 and October 2022 in the Web of Science, PubMed, Scopus, PsycInfo, ProQuest, Poplin and Google Scholar databases. Girl child marriage is defined as a marriage under the age of 18. In this study, the CASP evaluation checklist was used to collect data. Two independent reviewers reviewed all articles.

**Results:**

A total of 34 eligible qualitative articles were included. The most salient causes of child marriage among girls include low skills and knowledge, internal and external beliefs and motivations, and physical advantages at the individual level. Family characteristics and structure contribute to child marriage at the interpersonal level, while environmental and economic factors play a role at the community level. Social factors and cultural norms, as well as the shortcomings and weaknesses of legislation, are also contributing factors at the society level.

**Conclusion:**

The results showed that cultural beliefs supporting gender inequality and economic status were the most important causes of child marriage. These results can help policymakers and decision-makers implement strategies to reduce gender inequality to prevent child marriage.

**Supplementary Information:**

The online version contains supplementary material available at 10.1186/s12889-023-17626-z.

## Background

Girl child marriage (GCM) is defined by the United Nations Children's Fund (UNICEF) as a marriage that occurs before the age of 18 [1]. UNICEF reports that the rate of GCM worldwide has decreased in recent years [2]. Yet globally, nearly 15 million girls under the age of 18 are married each year [3]. Today, approximately 750 million women are married as children, and this number will remain unchanged until at least 2030 if progress is not accelerated [4]. This phenomenon is widely visible. The phenomenon of GCM can be seen across a wide range of contexts. About 37% of global GCM occurs in sub-Saharan Africa, 30% in South Asia, and 25% in Latin America [5]. This practice also occurs in certain European countries [6–8].

Early marriage (EM) is not only a violation of children's fundamental rights, but also detrimental to women's health and public health. It has raised growing concern about its potential impact on population health [9, 10]. Studies show that girls who marry under the age of 18 have less control over their fertility [4, 11], limited access to contraception [12, 13], a higher likelihood of unintentional pregnancy , and an increased risk of birth complications that can result in death [14, 15]. They also suffer from other related health problems, such as domestic violence [16, 17], sexually transmitted diseases (STDs) [18, 19], and psychological problems [20, 21].

Although the international community and governments are becoming more aware of the harmful effects of GCM, efforts to eradicate it are still limited [22, 23]. This issue challenges the achievement of the 2030 Sustainable Development Goals (SDG) [24]. GCM is a multidimensional problem and is synergistically affected by several factors, and its solution requires a holistic perspective [25]. UNICEF also emphasizes that the causes of GCM cannot be examined from only one perspective, and strategies should be developed - in addition to key drivers - with regard to social and political factors, communities, families and girls [26]. The Socio-ecological Model (SEM) is a multi-level approach that is used to better understand systemic effects in health-related issues and identify intervention points. This model is able to identify the driving factors of GCM at the individual, interpersonal, social and community levels [27]. Determining the determinants of GCM at different levels facilitates the design of programs and preventive interventions and reduces the burden of EM and its damage.

Quantitative evidence shows that poverty status, low educational attainment, rural living, and religion are associated with CM [28]. Some studies have investigated the factors that contribute to CM. The systematic review by Kohno examined 12 studies (2008-2018) exploring the causes of CM. He identified six key factors in CM, including human insecurity and conflict, legal issues, family values and circumstances, religious beliefs, personal circumstances, beliefs and knowledge, and social norms [29]. Research conducted by Pienar Duro (2005-2020) identified the occurrence of early and forced marriages and concluded that cognitive, emotional, behavioral, and cultural factors contribute to these marriages [30]. In a systematic review, Feyissa (2023) investigated the efficacy of interventions aimed at reducing child marriage (CM) and teenage pregnancy. There are five categories of interventions, including: (a) creating educational assets, (b) developing life skills and health assets, (c) building wealth, and (d) fostering community dialogue. They emphasized that systematically implemented scholarship and community dialogue interventions are consistently effective in various settings [31].

Although the aforementioned studies have provided valuable information about the driving factors of CM and the process leading to it, it appears that by incorporating a broader range of studies conducted in this field and identifying the various driving factors contributing to CM, the existing gaps in previous research can be addressed. Therefore, the present study was conducted with the aim of analyzing and interpreting social-ecological factors influencing GCM over a 22-year period (2000-2022). This was done using a meta-synthesis approach and a conceptual framework. Identifying the underlying factors that contribute to GCM at various levels is crucial for designing effective programs and preventive interventions. This approach helps alleviate the negative consequences of EM and reduces its overall impact. This study answers the main question, what are the key factors behind GCM at the individual, interpersonal, community, and society levels derived from the findings of previous studies?

## Method

### Design of study

This qualitative review was conducted in accordance with the Preferred Reporting Items for Systematic Reviews and Meta-Analyses (PRISMA) guidelines [32]. We used the Enhancing Transparency in the Qualitative Research Synthesis Report: (ENTREQ) Statement to prepare the paper [33]. The present study is registered in the PROSPERO system with the code CRD42022377071.The meta-synthesis technique was used to address the following research questions. The meta-synthesis technique was used to address the following research questions.

a What are the individual factors contributing to GCM?
b What are the interpersonal factors contributing to GCM?
c What are the community factors contributing to GCM?
d What are the society factors contributing to GCM?

### Information sources and search strategy

We used a pre-planned search strategy and retrieved all studies published in English between January 2000 and October 2022 in seven electronic databases including Web of Science, PubMed, Scopus, PsycInfo, ProQuest, and Poplin. We retrieved studies from 2000 to 2022. While traditions are fading and technology has solved many health-related problems, the phenomenon of child marriage still persists.

Haddaway suggested that the first 200-300 records of Google Scholar are useful for finding gray literature. Therefore, we also included the first 300 records of Google Scholar [34]. Search terms contain medical topics (MeSH), free words and selected keywords. We searched for all qualitative articles on CM using keywords. These keywords included;

"child marriage", "early marriage", "spouse child", "teen* marriage", "adolescent marriage", "child bride", "forced marriage", "interview", "focus group*", (group* and focus),"case stud*" observ*, view*, understand*, beli, feel*, custom*, percep*, "sensory process*", (processing and sensory), opinion*, attitude*, sentiment* ,(research and qualitative), qualitative

Two authors performed the search independently. Endnote software was used for data management and MAXQDA software was used for meta-analysis.

### Selection process inclusion and exclusion criteria

The purpose of this study is to determine the causes of GCM. Therefore, any qualitative research examining the reasons behind a phenomenon, including ethnographic research, grounded theory, and content analysis, is included in this review. All observational studies, mixed methods, reports, systematic reviews, trends, and studies reporting on the causes of CM among boys were excluded from the study. More details are shown in Table S1.

### Identification and selection of studies

We removed duplicate articles. Then, the titles and abstracts of the remaining studies were screened. The following is a comprehensive review of the study's full text in order to fulfill the study's objectives. The requirement for including articles was a 100% agreement between the two researchers. If there were a disagreement during the study review, a third reviewer would resolve the issue.

### Risk of bias assessment

The quality of selected studies was assessed using the Critical Appraisal Skills Program (CASP) for qualitative research. The checklist consists of 10 questions and is comprehensive, easy to understand, and widely used, making it a valuable tool [32]. The first two questions are used for screening purposes, inquiring about the study's objectives and the suitability of the method for achieving those objectives. If the answer to both questions is yes, the remaining eight research evaluation questions are asked. These questions include aspects such as study design, recruitment strategy, data collection, the relationship between the researcher and participants, ethics, accuracy of data analysis, clear reporting of results, and their implications. Two researchers (AP, MM) independently read, reread and evaluated the papers.

### Data extraction

Two researchers (AP, MM) independently extracted the data in accordance with the study's objectives. In addition to discovering the causes of GCM, detailed information from the articles was extracted. This included the author, year of publication, country, participants, sample size, research design, data collection methods, objective, and theme/main concept. Any disagreements between the authors regarding study eligibility were resolved through consultation with the third author.

## Analysis

**Fig. 1:**
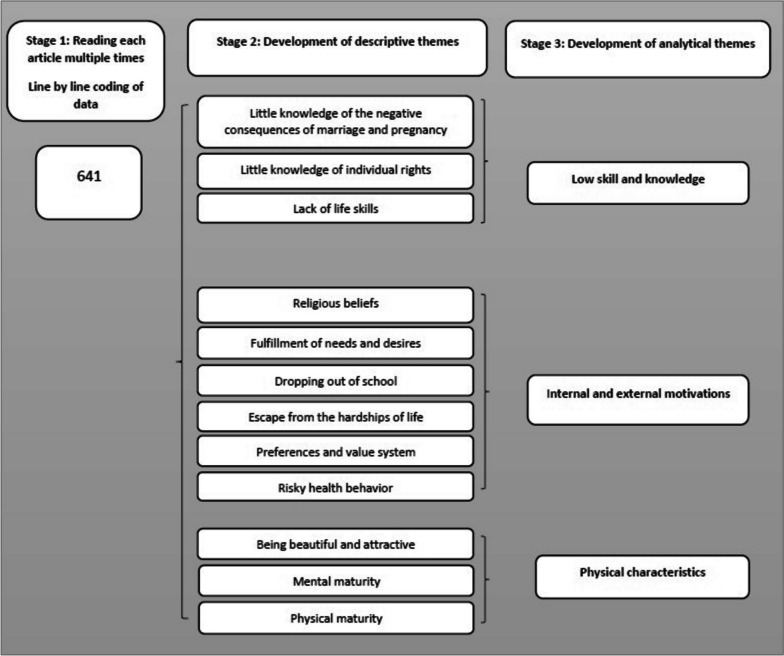
The process of synthesis of findings

The current study followed Thomas and Harden's (2008) approach to synthesizing qualitative research findings [35]. Thematic synthesis was chosen as the qualitative evidence synthesis method because of its usefulness in providing information about [36]. The meta-synthesis began by reading each article multiple times and reflecting on the data in an attempt to answer the research question of the study. All text under the headings ' Results' or 'Findings' electronically extracted and entered using MAXQDA computer software. We performed data synthesis in three steps. Steps 1 and 2 involved coding the text and developing descriptive themes or sub-themes. The results of the study were extracted and summarized based on the research questions. As suggested by Thomas and Harden (2008), in order to avoid imposing an a priori framework on the findings implied by the research question, these were set aside [35]. Thus, the process evolved from the study's results to a thematic analysis. These texts were entered into MAXQDA software, and each member of the research team independently coded each line of text based on its meaning and context. Most of them used more than one code for classification. The composition process began simultaneously. We identified similarities and differences among the codes and organized them into a structured format. New codes were created to capture the meaning of the original code groups. In the third stage, the analysis topics or themes are created. In this step, the descriptive themes that emerged from the deductive analysis of the research findings were used to address the research question that had been temporarily postponed. This process resulted in a structure of twenty-two subthemes. The synthesis process of individual factors is shown in Fig. 1.

## Results

**Fig. 2:**
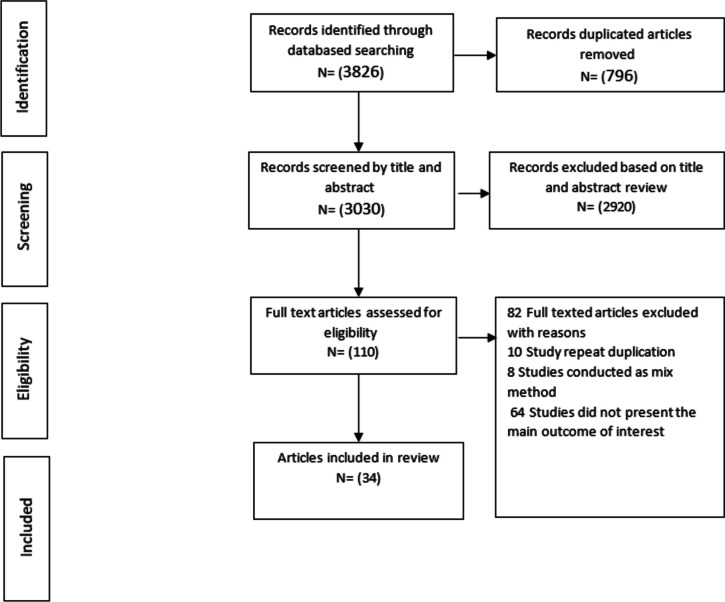
PRISMA flow chart diagram describing selection of studies for Meta-synthesis on cause of child marriage

The initial search of the database resulted in 3,826 articles. After removing duplicates, titles and abstracts, and full text screening, 34 articles were included in this meta-analysis in checking the reference lists of included studies, no other studies were found (Fig. 2).

A total of 34 studies were identified in the current review. In total, 14 of the included articles had been conducted in the Middle East [37–49], 9 in Africa [50–58], 8 in South Asia [59–66], 2 in Europe [8, 67],1 in the United States [68], and 1 each in Ethiopia and India [69]. The number of participants ranged from 8 to 300 people. Approximately 900 people participated in the study Table 1.

**Table 1:** characteristic of included studies in meta-synthesis

	**Author/year**	**Country**	**Participant**	**Sample Size**	**Study Design**	**Data Collection**	**Setting**	**Objective**	**Main theme/major concept**
1	Chowdhury/ 2004 [59]	Bangladesh	Villagers, NGO government officials, Union Parisad , teachers, Religious leaders	96	Participant observation,	Discussions, interviews	Rural	Reasons and consequences of CM	Social values, poverty, local beliefs, control a woman’s Personality, desire, dowry, necessity for guardians Provocations by male youths, girls’ beauty, shame, property
2	James/2010 [50]	Nigeria	Teenagers (both male and female)	300	Ethnography	FGD interviews	Rural/urban	Socio-cultural contexts for marriage pregnancy and childbearing	Parental pressure ,social norms, Individual needs, Desires, economic survival, connection, With wealthy and powerful individuals, Domestic help and guaranteed support
3	Matlabi/2013 [37]	Iran	CB, teacher. Rural population, parent. Married student	60	Content analysis	FGD interviews	Rural	Cause of the CM	Village culture and social pressure, Economic, lack of awareness of CM, Negative attitude toward education, Tend to marry young boys in early ages, Freedom from rigid rules of parents, Applying to girls, lack of access to school
4	sabbi /2013 [51]	Morocco	Stakeholders, NGO, teachers legal professionals, ,government representatives	22	Content analysis	Interviews	Urban	Occurrence of child and forced marriage	The legal and social divergence, legislation, education, economic
5	Nasrullah /2014 [38]	Pakistan	CB	20	Content analysis	Interviews	Slum areas,	Women’s knowledge and attitude towards CM	Culture and community perceptions, varying interpretation of religion, protecting family honor
6	Vang /2014 [68]	American	CB	12	Content analysis	Interviews	^a^Hmong	Socialization processes of CM	Family Socialization Processes, Individual Insight and Perspectives
7	Montazeri/2016 [39]	Iran	CB	15	Content analysis	Interviews	Urban	Determinants of CM	Family structure, Low autonomy indecision-making, Response to needs
8	Segal/2016 [67]	Israel	CB	10	Content analysis	Interviews,	Rural	Women's perspective on advantages and disadvantages of CM	Freedom and overall independence, solve attachment Poverty, violence.
9	Syamsidah/ 2016 [39]	Indonesia	Parent, community leaders, educators ,religious leaders	5 families	Phenomenology	Observation, interviews	Coast sitter	Background family life and the factors that cause CM	Background Family Life
10	Mangeli/2017 [42]	Iran	Adolescent mothers	16	Content analysis	Interviews	Rural/urban	Factors that encourage CM and motherhood	Internal External incentives, External incentives
11	Mourtada/2017 [40]	Lebanon	CB, women, parent ,key informant, stakeholders	20	Phenomenology	FGD, interviews	Syrian refugee	Influence factors To recommendations on decrease CM	Challenges faced by refugees, Change in marriage practices, Knowledge about negative, Consequences of child marriage, Current services
12	Iustitiani/2018 [62]	Indonesia	CB	8	Content analysis	Interviews	No	Supporting factors and the consequences of CM	Educational ,Social, ashamed, economic
13	McDouga/2018 [69]	Ethiopia, India	CB	105& 100	Content analysis	Interviews	Rural	The pathway of marital decision-making and reasons leading CM	Initiation, negotiation and final decision-making major concept
14	Muhith/2018 [61]	Indonesia	Community leaders, CB	24	Content analysis	Interviews	Rural	Factors driving and impact CM	Economic, education, parents and customs factors
15	Stark/2018 [52]	Tanzania	CB, key informants, mothers	171	Content analysis	Interviews	Urban	The motivations early marriage	Economic, education, sexual relation
16	Aleksandrova/2019 [8]	Bulgarian	CB	13	Content analysis	Interviews	Rural/urban Roma women	Factors influencing the process of CM	Social stressors in childhood ,Parents’ attempts to keep their daughters Pure, Getting married, Parental involvement in the process, Making marriage known to the community
17	Bhandari/ 2019 [66]	Nepal	School girls	60	Content analysis	Interviews	Urban	Influencing factors CM	Relationship between Dowry and Marriage Education, Qualities of a Good Husband
18	Cislaghi/2019 [53]	Cameroon	Men and women	80	Ethnography	FGD, interviews, Observations	Rural	The norms of CM	Girls’ age at marriage, Marriage negotiations and Wedding procedures, Respectable women marry early, Respectable women do not have premarital sex, Girls’ voice and parents role
19	Dean/2019 [41]	Sudan	CB, mother, father, key informant	83	Content analysis	Interviews	Rural	Drivers of CM	Moral and religious values, gendered social norms and relations Structural and social barriers to education, preferences for kinship And intervillage marriage partners, limited autonomy ,decision-making
20	Judy/2019 [43]	Somalia	CB, community elders, religious leaders	13	Content analysis	Interviews	Rural	Community perception and effective strategies on forced marriage	Push factors Pull factors
21	Kohno/2019 [63]	Malaysia	CB	22	Content analysis	Interviews	Rural	Reasons for CM	Immaturity in decision-making, poverty and religious and cultural norms
22	Kohno/2020 [64]	Malaysia	CB	22	Content analysis	Interviews	Rural	The factors leading to CM	Health risk behavior, family poverty, CM as fate, and Family disharmony.
23	Lebni/2020 [44]	Iran	CB	30	Content analysis	Interviews	Rural/urban	Social determinants of CM	Economic factors, sociocultural factors, individual factors , family factors, structural factors
24	Madut/2020 [45]	Sudan	Male and female	91	Grounded Theory	FGD, interviews	Urban	Socioeconomic factors influence on CM	Social and economic factors, social norm Traditional marriage ,gender roles
25	Elnakib2021	Egypt	CB girls, ,parent, Community Leaders, Health Providers, Humanitarians, Legal experts	72	Content analysis	FGD, interviews	Syrian refugee	The factors leading to CM	Disruptions to girls’ education, protection concerns, and livelihood insecurity, customs and arrangements, Economic insecurity, Culture and traditions, Schooling-related drivers, Gender norms
26	Mirzaee/2021	Iran	Girls ,key-persons, key-informants, , and mothers	179	Content analysis	FGD, interviews	Rural	The factors leading to CM	Social stigma, fear of stay single, the desire, parents concern The value of virginity, parents unawareness, fatalistic approach, Disappointment with continuing education, legislation, religious beliefs, The financial capability of families, the monotony of social life
27	Mrayan/2021 [47]	Jordan	CB	36	Content analysis	Interviews	Rural	Lived Experience of CM	Feeling remorse about getting married early, Loss of Authority and feeling powerless, Reasons behind CM, Feeling pressure to have the first baby, CM And pregnancy health consequences, The positive aspects of an CM
28	Neema/2021 [55]	Uganda	CB, Teenage,, parents, leaders/officials ,key informant	108	Ethnography	FGD, interviews, observations	Rural/urban	To explore underlying drivers of CM	Poverty and survival strategies, Socio-cultural beliefs and norms School dropout
29	SchaffnitI/2021 [56]	Tanzania	CB, parents, young men, community leaders	33	Content analysis	Interviews	Semi urban	Views of CM	Culture, schooling dropping out, Damage reduction, poverty
30	Susilo/2021 [65]	Indonesia	CB, parent	48	Phenomenology	Observation, interviews	Rural	Perceptions and impacts of CM	matchmaking, to avoid fornication before wedlock, to avoid out-of-wedlock pregnancy, CM due to economic factors
31	Baraka/2022 [57]	Tanzania	CB, parent	39	Content analysis	FGD Interviews,	Semi-urban	Drivers of CM	Economic benefits, Girls' decision to create opportunity, Men lure
32	Maghsoudi/2022 [48]	Iran	CB	14	Grounded Theory	Interviews	Urban	Paradigm model of the phenomenon of CM	Absence of family as a safe haven
33	Bozorgi/2022 [49]	Iran	CB	20	Phenomenology	Interviews	Urban	Experiences and the consequences of CM	Causes of CM, concerns and negative feelings, Exposure to violence, consequences of CM
34	Tewahido/2022 [58]	Ethiopia	CB, parent	158	Content analysis	FGD	Rural	Social norms on CM	Empirical ,normative expectations

^a^Hmong population in the United States are one Southeast Asian ethnic groups

*CB* Child bride, *FGD* Focus group discussion, *CM* Child marriage

### Risk of bias assessment

All studies that answered affirmatively to the first two questions of the CASP assessment checklist were included. No studies were excluded based on the CASP score. We discussed the results of the quality assessment in regular meetings, resolved our differences, and reached an agreement on 34 studies. In most studies, the study design (*n* = 33), recruitment strategy (*n* = 31), data collection methods (*n* = 29), ethical issues (*n* = 27), data analysis (*n* = 23), clear findings (*n* = 32), and valuable results (*n* = 31) were explained. In only 8 studies, the relationship between the researcher and the participants was adequately considered Tables S3, S4.

#### Thematic synthesis

**Fig. 3:**
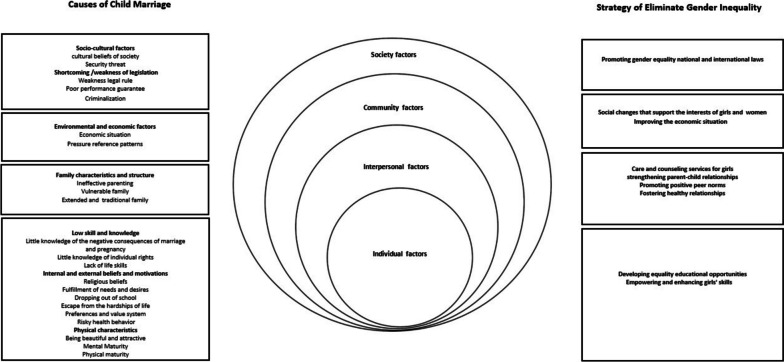
Causes of GCM and proposed strategy to eliminate gender based on Socio- ecological Model

We categorized the socioecological factors of GCM into four levels: The synthesis and contribution of each study are shown in Table 2. Three themes at the individual level (low skills and knowledge, internal and external motivations, physical advantages), one theme at the interpersonal level (family characteristics and structure), one theme at the community level (environmental and economic factors), and two theme at the society level (social and cultural factors, deficiencies/weaknesses of legislation). More details were shown in Table 3 and Fig. 3.

**Table 2:** Themes and review findings

**Theme/Sub theme**	**code**	**quotation**
***Individual factors***		
**1. Low skill and knowledge**		
1.1 Little knowledge of the negative consequences of marriage and pregnancy	Insufficient knowledge about physical, sexual, psychological and social problems, Insufficient information about the consequences of marriage and pregnancy	I didn’t think to that level. Because I thought marriage wasn’t… it was just like a game. I just followed. I knew nothing. Even when I was pregnant, I didn’t know what to eat, how to do family planning. I didn’t know. That’s why I gave birth to my three children in three years. Small birth spacing [laugh]’. (No. 20, married at 17 years old) [63].
1.2. Little knowledge of individual rights	Inadequate knowledge of girls and women about marriage laws, Inadequate knowledge of sexual rights	Women do not know enough about their basic sexual and reproductive rights [51].
1.3.Lack of life skills	Inability to negotiate, Improper decision making, problem solving, Critical Thinking	Had never thought about marriage or the guy who I was going to marry. I couldn’t make decision appropriately. It bothered me so much because I was not ready for marriage and had to marry while still being a kid [39].
**2. Internal and external motivations**		
2.1.Religious beliefs	Following the traditions of the prophets, pleasing God, obeying parents, respecting parents, and accepting the fate	Marriage is of God, whether a girl marries early and gives birth or not if she is destined to face problem she will definitely do so. There are cases of older women who still have pregnancy and child birth complications, and if God wishes, a girl that marries early can deliver safely without any complications. All these are the wonders of God, so to me teenage marriage is not a serious problem because in another way it helps in maintaining sanity in the society since girls are so corrupt and cannot keep them selves pure these days [50].
2.2.Fulfillment of needs and desires	Needing freedom, independence, companionship, enjoyment of life, love and infatuation, desire for sex, dream of a romantic life, gaining prestige, showing aristocratic life	No one convinced them [to marry]. They decided that themselves. [A] friend of mine was tempted to be with her lover and they got married without even telling her parents. She eloped with her lover. They decided to get married because of their own desire [56].
2.3.Dropping out of school	Lack of educational resources, declining educational attainment, lack of schools, male teachers	my brother doesn’t let me go to further city for continuing my education because their people aren’t good and they cause harassment for us [42]
2.4.Escape from the hardships of life	life's challenges and hardships, the weight of responsibilities, societal norms, and parental strictness	It was a way out for me. My dad very abusive, not only that, I had to come home from school every day and watch my nieces and nephews, cook, and my parents never let me do anything [68]
2.5.Preferences and value system	Spouse's job, the spouse's superior status, and the spouse's personal status	My dad told me, the boy is a nice person to marry and I shouldn’t reject his proposal [39]
2.6.Risky health behavior	Alcohol and drug misuse, Unprotected sex	enjoyed myself.... First, I was in jail because my mom was suspicious about my behavior, and I always talked back. So, she wanted to check, and she told the police to take me. They checked my urine and took me to the drug rehabilitation center [64]
**3. Physical characteristics**		
3.1. Being beautiful and attractive	Beauty, Being attractive to men	I was being harassed by a young hoodlum because of my attractive appearance. So I was married off at an early age [59].
3.1.Mental maturity	Understanding emotional maturity in relation to age, decision-making ability	I wanted to marry because l thought if I get married, I will become more responsible. I think I know more than my friends who are still single. I have a sense of primacy. I feel I ammature than them [39].
3.3.Physical maturity	Start of menstruation, Being large	In this area, [marital readiness] is not decided by age. We see whether she has matured enough to manage her home after marriage. More of the estimation is based on her physical appearance than her age. At that time she can be 11 or 12 years of age. Sometimes one can make an engagement at the age of 8 and marry her when she reaches 12. –Male decision-maker for girl whose early marriage was delayed/cancelled (relationship: father) [69]
***Interpersonal factors***		
**4. Family characteristics and structure**		
4.1. Ineffective parenting	Concern, Irresponsibility, Incompatibility Parents, Ignorance Of The Child's Interests, Lack Of Intimate Relationships	My father had elementary education. My mother was totally illiterate. They knew nothing. They thought if I got married sooner I would be happier [44].
4.2. Vulnerable family	Domestic Violence, Parental Addiction, Divorce, Loss Of A Parent, And Absence Of A Guardian	My father was an addict. My mother used to say that no one would marry the daughter of a drug addict [44].
4.3. Extended and Traditional family	The Involvement Of Relatives, Extreme Prejudices Of The Family, Dominance Of The Father In The Family (Patriarchy), The High Dimension Of The Family/Several Children, Decision-Making By Parents	It was my father's words, don't ask me if you want to get married or not [48].
***Community factors***		
**5. Environmental and economic factors**		
5.1. The economic situation	Poverty, financial burden, weak family financial support, inability to meet basic needs, challenging living conditions, poverty caused by natural climate change, lack of employment opportunities	We were very poor. I had got some education in school. I had seven sisters. After the birth of my three sisters we got a brother. But after a few days he died. My mother got pregnant often, hoping for a son [59].
5.2.Pressure reference patterns	Approval from peers, encouragement from relatives, media advertising	I think if the girl is approved to get married by her parents and my parents, it means that she is ready to get married. We don’t measure woman’s readiness for marriage by her age, but by her ability to have children and manage her family. I wouldn’t marry a lady of whom my parents and relatives do not approve. I can’t divorce my spouse without their consent regardless of the type or nature of the problem [45].
***Society factors***		
**6. Socio-cultural factors**		
6.3. cultural beliefs of society	Avoiding dowry, being compatible with young girls, delaying marriage, maintaining virgin, preserving reputation, exchanging goods, settling blood ties, peace, consolidating friendship and kinship, avoiding sin, maintaining similarity with relatives, social value, prohibition of premarital sex , magic, preserving family property, avoiding social stigma	It is recommended that a girl be married off before the age of 18, preferably between the ages of 15 or 16, in order to avoid shame befalling the family”, said one community elder. When a girl delays marriage, she is considered flawed and is thought to bring bad luck to her family [43].
6.4. Security threat	War and displacement, kidnapping, extortion, physical violence, threats	Fear of insecurity is a major factor. They are marrying early because of al Sutra. We have war. Many women are afraid of being raped, and if a married woman is raped, she is more likely to be forgiven by her husband but if an unmarried woman is raped, it will destroy her life [40].
**7. Shortcomings/weaknesses of legislation**		
7.1. Weakness of legal rules	Not prohibiting early marriage, The possibility of an illegal marriage	I didn’t want to get married, I didn’t know anything about marriage, but my family forced me to get married. My aunt (mom’s sister) tried to stop it, she even talked to a lawyer but she could do nothing because the law does not give the girls any rights [44].
7.2. Poor performance guarantee	Lack of legal support structures, Poor supervision	We as an NGO follow up cases, where there are reports about parents giving in their teenage girls for marriage and they are arrested, however the biggest problem sometimes comes from the police side, the police accept that these two parties should come and negotiate, and settle their cases outside the court [55].
7.3. Criminalization	Criminalization of sexual relations outside of marriage, Criminalizing relationships between boys and girls	We have to suggest that our girls get married, you know? From the age of 16, after puberty. And, it sounds like we are trying to save the society so that they don’t accidentally become pregnant and all that [63]

**Table 3:** Matrix of included studies and identified themes

	**Low skill and knowledge**			I**nternal and external motivations**						**Physical characteristics**		
Author	Little knowledge of the consequences of marriage and pregnancy	Little Knowledge of individual rights	Lack of life skills	Religious beliefs	Fulfillment of needs and desires	Dropping out of school	Escape from the hardships of life	Preferences and value system	Risky health behavior	Being beautiful and attractive	Mental maturity	Physical Maturity
1				√	√		√	√	√	√		√
2				√		√						√
3	√		√	√	√	√	√	√		√		
4	√	√			√	√						√
5	√			√		√		√				
6	√				√		√		√			√
7	√		√	√	√	√	√	√			√	
8					√	√	√	√	√		√	
9					√	√	√		√			
10	√			√	√			√				√
11						√		√				
12	√		√	√	√	√	√		√			
13				√	√	√					√	√
14					√	√	√		√			√
15					√	√	√	√	√			
16				√	√	√	√		√			
17				√		√		√				
18				√	√							√
19			√	√		√		√				
20				√	√	√	√					√
21	√		√	√	√	√	√	√	√			√
22	√			√	√	√	√		√			
23	√			√	√	√	√	√		√		√
24			√		√	√		√	√			√
25				√		√	√					√
26				√		√						
27	√			√	√	√	√					
28			√	√	√	√	√		√			√
29					√	√	√		√			
30				√	√	√			√			
31						√	√		√	√		√
32			√	√	√	√	√					
33				√	√		√					
34				√	√	√						√

	**Family characteristics and structure**			**Environmental and economic factors**		**Socio-cultural factors**				
Author	Ineffective parenting	Vulnerable family	Extended and traditional family	Economic situation	Pressure Reference patterns	cultural beliefs of society	Security threat	Weakness of legal rules	Poor performance guarantee	Criminalization
1	√	√	√	√		√	√			
2	√		√	√	√	√		√		
3			√	√	√	√				
4			√	√		√		√		√
5			√	√		√				
6		√	√		√	√				
7			√	√	√	√				
8		√		√		√				
9			√	√	√	√			√	
10	√	√		√	√	√				
11				√		√	√			
12				√	√	√		√	√	
13		√	√	√	√	√			√	
14	√	√	√	√	√	√				
15			√	√		√				√
16	√	√	√	√		√		√		
17	√	√	√	√	√	√				
18	√		√			√				
19			√	√	√	√	√			
20			√	√	√	√	√	√		
21	√		√	√	√	√		√		√
22	√	√	√	√		√				
23	√	√	√	√	√	√		√		
24	√	√	√	√	√	√		√		
25				√	√	√	√			
26	√		√	√	√	√	√			
27	√		√	√	√	√		√		
28	√	√	√	√	√	√			√	
29	√		√	√		√	√			√
30	√		√	√	√	√				√
31		√	√	√	√	√		√		√
32	√	√	√	√		√		√	√	
33		√	√	√		√				
34			√	√	√	√

The most salient individual factors of GCM include low skills and knowledge, internal and external thoughts and motivations, and Physical characteristics.

### Theme 1: low skill and knowledge

#### Little knowledge of the negative consequences of marriage and pregnancy

Most of the participants did not have enough information about the consequences of marriage, pregnancy and childbirth. They admitted that they had no idea what to expect when they entered into marriage. They were unprepared and unaware of their new responsibilities as wives and mothers, including housework and taking care of family members [37–39, 42, 44, 51, 62–64, 68]. “I was really concerned about pregnancy and childbirth." If I had known that marrying early would lead to such physical and mental problems and ruin my life, I would never have gotten married” [44]. Society appears to be indifferent to girls' lack of knowledge about marriage and pregnancy. In Morocco, discussions about sexuality are considered social taboos that people, especially young individuals, tend to avoid [51]. One of the informants said, “We do not discuss these matters because of the social stigma. Many boys and girls do not receive comprehensive sex education” [51]. In Iran, there is insufficient media coverage [44] and a lack of counseling and decision-making services for girls [39].

#### Little knowledge of individual rights

Girls are not aware of marriage laws and sexual rights. Sometimes, the law is disregarded due to a profound lack of awareness among girls and women regarding their rights [51]. One of the informants said, "In rural areas, many child marriages go unreported in the Ministry of Justice's statistics." These marriages take the form of a simple ‘Fatiha' (declaration), remain unregistered, and transform girls into married women without their awareness [51].

#### Lack of life skills

A lack of independence in decision-making, caused by deficiencies in life skills such as decision-making, problem-solving, negotiation, and critical thinking, expose girls to EM. Some girls felt that they were unable to make informed decisions about marriage due to their inability to anticipate the potential outcomes of their choices. Hence, they accepted the decisions made by their parents [37, 39, 41, 45, 48, 55, 62, 63].“I am not mature enough to judge people's thoughts and behavior. I didn't feel good about getting married because I didn't know my fiancée very well. So I left everything completely to my family” [39].

### Theme 2: internal and external motivations

#### Religious beliefs

James highlighted the inflexible beliefs that prevail in rural society in northern Nigeria. Influenced by these beliefs, teenagers supported the idea of early marriage. One of the girls said, “I support teenage marriage and having children. It's not a problem. Marriage is a source of pride for women, and it is expected for every Muslim and Christian girl to get married.” Adolescent boys also viewed marriage as a means to fulfill God's commandments and to follow Western traditions [50]. Girls in the Muslim community of Malaysia also believed that fate decreed that they should marry at a young age. Marriage is an inevitable decree of God that must be obeyed [64].

#### Fulfillment of needs and desires

Adolescents are in a crisis during their teenage years. Neglecting them and their needs can lead to engaging in risky behaviors and EM [70]. Gaining independence and freedom, seeking significance, the desire to form relationships with the opposite sex, and the longing for a life partner are some of the factors that motivate girls to marry at a young age [71, 72]. In addition, in Muslim countries, the sexual needs of girls can only be fulfilled through marriage and within the framework of religious and customary [73]. In the Kurdish regions of Iran, cultural and social beliefs contribute to a more favorable perception of young brides and greater support for them. As a result, they often receive larger dowries. “In our region, when we are younger, there is often more emphasis on dowry, but I did not want to get married at all” [44]. In Morocco, many girls aspire to marry someone residing in Europe. They have idealized images of a wonderful life abroad in their impressionable minds. “The marriage market acquired on a dimension, element, as many women aspire to marry marrying a Moroccan residing living Europe” [51]. Marriage may not always be in the best interest of a girl, but it can be a strategic choice for her, given the current situation.

#### Dropping out of school

In rural and impoverished areas, families cannot afford the direct costs of education such as tuition and books, as well as the indirect costs of transportation or accommodation at distant middle schools. Additionally, they were uncertain about future career prospects. In this case, the girls voluntarily dropped out of school to help with household chores [37, 47, 52, 57]. Also, the negative attitude towards girls' education, preservation of cultural values [37, 44, 55, 65], lack of interest in education [52, 56, 57], poor academic performance [55], war and displacement [40] have reduced the educational opportunities for girls and have provided grounds for EM . One of the Syrian refugees said, “We used to enjoy studying and getting married, but now we are affected by the Syrian war”. The situation has changed [40].

#### Escape from the hardships of life

The participants used marriage as an avoidance coping strategy to escape the difficulties of life. In most cases, poverty was seen as an integral part of challenging living conditions. Syrian refugees in Lebanon described their dire financial situation, living in small tents, in unsanitary and insecure conditions as difficult and exhausting. In order to escape these conditions, they resorted to marrying off their young girls [40]. In Somalia, environmental degradation and frequent particularly especially in rural along with and war, result in make girls becoming girls victims of early and forced marriages. One of the girls said, “Perhaps when I marry a wealthy older man, I will continue my education, as all my needs such as educational materials, school fees, and travel expenses will be covered by him” [43].

#### Preferences and value system

The characteristics of suitors, such as their occupation, social status, and personal background, were factors influencing the children's choice of spouse. They believe that suitable marriage opportunities should not be missed [37–42, 44, 45, 52, 59, 63, 66, 67]. Girls also welcomed the opportunity to marry a good suitor. “I feel very fortunate to have such a good, intelligent, and caring person in my life. I believe that girls should marry at a younger age, provided it's a suitable match “ [38].

#### Risky health behavior

Some of the girls in this study indicated that they had been involved in high-risk activities during their teenage years, such as drinking and using drugs with their peers [64]. Through a network of friends, they found a partner with whom they had sexual intercourse and became pregnant. In order to conceal shame and scandal, the family compelled the girl to enter into an immediate marriage in accordance with Sharia standards [8, 45, 52, 55–57, 59–65, 67, 68]. “We engaged in sexual activity before marriage, even though it was wrong. "Then I found out I was pregnant while I was working [64].”

### Theme 3: physical characteristics

#### Being beautiful and attractive

Young and attractive girls were more attractive to men [37, 44, 57, 59]. The desire of men to marry young and beautiful girls has created a fear within families that if their daughters grow up, no one will want to marry them, which would bring shame to the family [61, 69], Some boys deceive and have sex with them. “I'm a pretty girl, and a lot of guys were attracted to me”. I learned that if I don't get married, I might end up in a situation involving sex outside of marriage [39].

#### Mental maturity

Maturity is a sign of readiness for marriage. Some girls believed they were more sensible than their peers, so they agreed to get married [39, 67, 69]. “Marriage was very important to my family. My mother told me that I am no longer a little girl after getting married. I will become more mentally mature than before. I have more plans for the future” [39].

#### Physical maturity

Parental illiteracy and adherence to cultural norms expose girls to EM. According to religious and cultural standards, girls who have reached a certain age, started menstruating and developing breasts, are eligible for marriage. One of the girls said, “People think that when a girl grows up physically, she is ready to marry” [39].

The characteristics and structure of the family are the most important interpersonal factors that provide the foundation for GCM.

### Theme 4: family characteristics and structure

#### Ineffective parenting

Ineffective parenting jeopardizes the bond between parents and children. Sometimes this relationship is influenced by parental concerns. Families concerned about their child engaging in premarital sex may consent to the marriage of their daughters at a young age [8, 46, 49, 54, 66]. Sometimes, the parent-child relationship is influenced by the parents' irresponsibility [50, 59, 61], incompatibility [48], and ignorance of the child's interests [55]. In Ineffective families, children may have to compete with each other for their parents' attention or affection, which can also influence their decisions about marriage. One of the girls said, “Our family was not warm and strong. We lacked affection with my sister so much that we fell in love with cartoon characters. We wanted to marry and live with them” [48].

#### Vulnerable family

A family atmosphere is an important factor in shaping children's attitudes towards marriage. The family provides a safe haven for the children, where they can grow surrounded by the love and attention of their parents. In families that lack stability, children may experience harmful effects such as CM, behavioral issues, emotional disturbances, and moral abnormalities. If children lose the support of their parents due to reasons such as the death of their parents, divorce, addiction, or violence, they may prefer to marry at a young age, or their parents may consent to their marriage due to behavioral indecisiveness. said one of the girls [48, 49]. One of the girls said, "I was thinking, 'God, can I get married one day and be free from this family?" [48].

#### Extended and traditional family

Extended and traditional families usually have a large population [44, 52]. The decision-making frameworks in these families are made by social norms and values, and all the decision-making processes are assigned to men [44, 45, 48, 51, 59]. In these families, the father's relatives can decide on the marriage of daughters as much as the father [41, 51]. “My father did not ask me if I want to get married or not. (My father decided on my marriage)” [48].

Community factors related to GCM included environmental and economic aspects.

### Theme 5: environmental and economic factors

#### The economic situation

Most of the participants cited the challenging economic situation and poverty as reasons for marrying at a young age. Poverty, financial burden, lack of family support, inability to meet basic needs, challenging living conditions [39, 42, 45, 47, 52, 54, 56, 57, 59, 61, 63–65], poverty resulting from climate change [43], and lack of employment opportunities [51] are all factors that contribute to EM. Some of them were not passive victims of their life circumstances, but actively trying to solve the problems in their lives. They decided to get married. “If I get married, I will feel like a bird leaving the prison. I will transform into a butterfly that soars toward freedom and happiness. "I will also assist my mother because she will have one less child to worry about” [67].

## Pressure reference patterns

Most of the participants considered GCM as normal. In these communities, neighbors, community elders, relatives and religious leaders encouraged parents to marry off their daughters at a young age. Here, girls had no choice but to accept marriage [42, 45, 66, 69]. In Morocco, [74] and South Sudan [45] grandfathers and grandmothers play a decisive role in arranging marriages for girls. Not accepting their offer is considered disrespectful. The media is often described as a “double-edged sword”. On one hand, it can help reduce CM by raising awareness about its harmful consequences [75]. On the other hand, it can also contribute to an increase in CM by portraying stimulating images and promoting sexual activity [65].

One of the men said, “I think that if a girl is approved by her parents and my parents for marriage, it means that she is ready for marriage. We do not measure a woman's readiness for marriage solely by her age, but rather by her capacity to bear children and effectively manage her family. "I will not marry a woman who is not approved by my parents and relatives” [45].

Socio-cultural factors and the shortcomings and weaknesses of legislation were the most prominent factors facilitating GCM within society.

### Theme 6: socio-cultural factors

#### Cultural beliefs of society

Most of the participants mentioned the beliefs that underlie CM. Some of these beliefs had religious roots, such as the preservation of reputation [59], while others had cultural origins, such as maintaining similarity with relatives [68]. In traditional societies, women's sexual purity is especially important, parents fear that their daughter will be involved in an emotional or sexual love. If their son or daughter is involved in an emotional or sexual relationship, a sinful act has been committed [41, 44, 65] and it is a shame for the family [38, 40, 43, 44, 47, 50, 59, 66], Even for the informed family, these types of activities are against social norms, and the family must face negative social sanctions [66]. In some cases, families consent to the marriage of girls out of fear of magic [59]. Despite all the worries about girls, they are a suitable tool for consolidating family relations [39, 41, 44, 46–48, 53, 61, 65], peace between tribes [43], blood lust [44] exchange for money [56], Exchange with cattle [55], and preservation of family property [47, 59]. “Pronatalism” is a salient social norm that encourages early marriage in Sudan. Men prefer younger women because they are perceived as more fertile [41]. It is believed that young girls have more sexual and reproductive power [59] and are better compatible with their wives [38], so the men looks for a young bride. “My husband used to be my boyfriend. When my family found out about this, they said that this relationship should be formalized. Then my husband proposed to me while we were both under 18 years old” [44].

## Security threat

Any factor that jeopardizes the safety of girls can contribute to the prevalence of GCM. Syrian refugees in Lebanon have identified feelings of insecurity and vulnerability to verbal or physical harassment while staying in unfamiliar and unsafe areas as one of the reasons for early marriage. Fear of insecurity is the primary factor. They marry early because of "Al Sutra" (the protection of the woman's honor or reputation). We are at war. Many women fear rape, and if a married woman is raped, her husband is more likely to forgive her. However, if a single woman is raped, it can devastate her life [40]. In some area, young girls are harassed by single young men or threatened with violence, such as rape, kidnapping, or acid attacks, when they reject marriage proposals. The family can address these threats and uphold its prestige by arranging marriages for their daughters [59].

### Theme7: shortcomings/weaknesses of legislation

#### Weakness of legal rules

The results showed that despite having legal knowledge and supportive laws and policies, harmful social norms overshadow them and have the potential to perpetuate CM. In some Muslim countries, such as Somalia [43], Bangladesh [76], and Iran [46], where the official laws are based on Sharia law, this legal system does not hinder GCM. According to their customs, girls are considered eligible for marriage after reaching puberty. In Somalia, where a triple judicial system exists, consisting of sharia, customary, and formal laws, the legislator does not possess the authority to intervene in the choice to marry through a traditional ceremony, as this is deemed acceptable under traditional laws [43]. In Morocco, where sex outside of marriage is illegal, the law effectively condones the crime [51], and in Malaysia, the "legitimate heir" law legitimizes CM. For example, if you are a legitimate heir, a valid marriage grants your children access to the family inheritance [63].

#### Poor performance guarantee

Participants pointed out legal loopholes, such as the mixed efficacy of laws in Ethiopia, as an example. Legal restrictions sometimes serve as a reason to prevent or delay early marriage, and in some cases, punishments related to legal violations have been recognized as deterrents from seeking legal help to delay marriage [69]. In Iran, there are no robust laws to prevent CM, and there is inadequate oversight of their enforcement. Civil organizations are also not sufficiently robust to support girls who do not wish to marry in childhood. said one of the girls: “In Iran, a large number of underage marriages occur every day, and there is little to no public outcry against it “ [44].

#### Criminalization

In some jurisdictions, sexual relations between unmarried individuals are considered illegal, and those who engage in such acts may face legal consequences [51, 63]. Most families, when faced with these circumstances (sexual relations between a girl and a boy), agree to the marriage of young girls [64]. Also, when judges are allowed to consider the best interests of children in marriage, it can also result in CM [51]. “When I was young, I engaged in premarital sex. So, we had to get married” [63].

## Discussion

GCM is a public health problem and one of the most evident instances of human rights violations. Every year, a large number of girls are victims of CM. GCM is the result of the interaction of many factors and occurs across countries, cultures, ethnicities, and religions. Globally, an increasing number of countries are acknowledging the detrimental effects of CM and are collaborating to strengthen laws against this practice. However, this practice persists due to complex underlying factors. The SEM is a useful tool for understanding the complexity and the relationships between factors associated with GCM. Using SEM, we identified seven main themes and 22 subthemes across four levels: individual, interpersonal, community, and society factors. We found that cultural traditions and beliefs have an important influence at all levels and are major factors in the prevalence of child marriage. The themes of “low skill and knowledge “, “internal and external motivations“, “physical characteristics”,” family characteristics and structure”,” environmental and economic factors”, ”socio-cultural factors”,” shortcomings/weaknesses of legislation” are emphasized in our findings.

The results showed that individual factors such as limited knowledge, internal and external motivations, and physical characteristics that predispose girls to EM are influenced by social norms and cultural traditions. The perceived status of women and girls in culture and society affects all aspects of their lives [44]. After birth, girls are often perceived as a burden to the family, while boys are seen as an asset [45, 59]. As a result, investing in or educating a girl child is often considered a waste of money because she is expected to eventually move to another man's house [50]. Girls' education is undervalued, and there is a focus on enhancing educational opportunities for boys [41]. Under the influence of cultural beliefs. Girls are not permitted to leave the village environment because doing so can result in the erosion and rejection of societal values. In fact, the prioritization of removing girls from school is aimed at preserving cultural values and family honor, regardless of the ability to pay for education [8, 37, 44, 51, 56]. By demonstrating the connection between education and CM, promoting girls' education is considered a strategy to reduce CM. India has seen a 38% decrease in CM over the last decade [77]. The geographical disparities in the prevalence of CM in Ethiopia are attributed to the expansion of girls' education [78]. The level of education reflects a person's maturity in terms of their ability to comprehend and respond to the environment and the knowledge available to them, making it easier for people to embrace and choose positive change [79]. Uneducated women are less actively involved in various activities that promote knowledge, such as reading materials, accessing service advertisements, and engaging in peer discussions. This lack of engagement makes them less aware of the detrimental effects of EM [80, 81]. Belief in fate [63], missing out on educational opportunities [40], overcoming life's challenges [44], and achieving prosperity and a better future [67] are also influenced by the cultural traditions associated with girls. The physical characteristics that result from biological processes are used to justify the marriage of girls within the cultural and religious framework [38, 39].

Family characteristics and structure were identified as the most prominent interpersonal factor. This is where it plays an important role in the formation of marriage rituals and family structure, allowing social and cultural norms to be preserved and passed down to the next generation. In some societies, social, economic, and livelihood issues are still influenced by traditional gender roles. In this context, men are expected to take on the responsibility of economic development and ensuring livelihood security, while women are expected to work at home, give birth, and take care of children. In fact, these discriminatory attitudes toward girls, which begin immediately after birth, are even more prevalent among impoverished families [45]. Most of the participants portrayed women as oppressed and marginalized by the traditional and religious patriarchal system at the family level. At times, this system dominates the family to such an extent that women feel like strangers to it and fail to comprehend it. The socio-cultural patriarchal context in Afghanistan has convinced women that violence against them by their male counterparts is acceptable and a form of love [17]. In many Muslim countries, premarital sex is culturally, religiously, and legally prohibited. Parents are concerned about their daughters engaging in emotional or sexual relationships, so they prefer them to marry at a young age [46, 63]. They believe that after marriage, they are no longer responsible for their daughter [44, 59, 66]**.** Encouraging girls to marry early when they are orphaned or have no competent parents to take care of them is also a way to control them and prevent them from engaging in sexual activity [59].

Environmental and economic factors were identified as the most important social factors related to GCM. Although in societies with low socio-economic status such as Bangladesh [82], India [83], Ghana [84], Ethiopia [85] GCM is considered an economic strategy to reduce the financial burden of parents, the powerful role of social norms should not be ignored. One concern among Syrian refugees in Lebanon is that exposure to Lebanese social norms, which seem to be more liberal than Syrian norms, may lead some parents to arrange early marriages for their daughters [40]. In northern Nigeria, in addition to the poor economic situation, pronatalist attitudes and opposition to foreign influences (or Western influence) reflect the strength of social and cultural norms and reproductive laws that support GCM. Poverty appears to have a synergistic effect on social norms and GCM decisions.

Socio-cultural factors and deficiencies/weaknesses of the law were introduced as factors related to GCM at the society level. It is worth considering that despite the wide geographic scope of CM, common cultural beliefs are prevalent among different regions. Maybe Swidler's theory (1986) can help us understand this phenomenon. Swidler defines culture as a toolbox of actions available to people, consisting of cultural collections such as worldviews, symbols, and stories. According to this approach, people do not necessarily act based on their values. Instead, they choose an appropriate action from the available options and then adapt their values to align with that action. The religious worldview appears to be the mechanism by which values are shaped, leading to the acceptance of the practice of CM [86].

This worldview has influenced the adoption of legal approaches in countries. Some countries have adopted the age of 18 for marriage, while others have set a lower age limit. In some cases, customary laws dictate the marriage age of girls, and the law has ceased to act as a deterrent [43]. In some regions, girls are permitted to marry at a young age with the consent of their parents or judicial authorities [46]. This has resulted in approximately 100 million girls around the world being denied the protection of national law, with the majority of them in the Middle East and North Africa [87]. Undoubtedly, when the law clearly specifies the minimum age for marriage, preventing CM is more achievable, but not necessarily less common. In societies where CM is viewed as a culturally acceptable way to safeguard girls from premarital sex and its potential consequences (such as unwanted pregnancy and sexually transmitted diseases), implementing, and monitoring the law becomes more challenging. It is essential to strike a balance between cultural and religious laws and norms to ensure that women's and girls' rights are protected while upholding moral values.

## Limitation

This study has several limitations. Firstly, we only included articles written in English, which means we may have overlooked articles written in other languages. Second, we attempted to access all of the articles, but we had to exclude the ones for which we did not have full text access. Third, the majority of the articles included in the synthesis are focused on Asia and Africa. We found few studies from Europe and the Americas, so we were unable to definitively address the causes of GCM in these regions.

## Conclusion

The results show that cultural beliefs and economic status are the most important factors influencing GCM. The results confirm that marriage is a social construct influenced by values. These values have been respected since ancient times and are still upheld by society and families. In some of these values, traces of gender attitudes can be observed. Beliefs are so powerful that they serve as the foundation for the establishment of laws and have a greater impact on parents' decision-making than economic factors [41]. In countries like Malaysia [88] and Indonesia [89] gender-based cultural norms are still influenced by religious and traditional beliefs, leading to a high prevalence of CM, despite economic development and high levels of education. All individual, interpersonal, community, and society factors influenced by these traditions appear to contribute to GCM. These traditions, in the context of modernity, can pose a significant challenge in attaining sustainable development goals. However, solving this problem requires special skills and precision. Our findings showed that the use of the SEM is a powerful tool in uncovering the underlying factors contributing to CM in various contexts. This model can assist policy makers and decision makers in gaining a comprehensive understanding of the causes of CM and in developing targeted strategies that address the specific needs of the affected population and community. The development of gender equality as a strategy seems to be effective in preventing GCM. These strategies include developing equal educational opportunities, empowering and enhancing girls' skills at an individual level, establishing a comprehensive network of care and counseling services for girls, strengthening parent-child relationships, promoting positive peer norms and fostering healthy relationships at an interpersonal level, advocating for social changes that support the interests of girls and women, and improving the economic situation in community level. Promoting gender equality national and international laws at the social level, play a crucial role in safeguarding the rights of girls.

### Supplementary Information

**Additional file 1:** **Table S1.** Enhancing transparency in reporting the synthesis of qualitative research: the ENTREQ statement. **Table S2.** Inclusion and exclusion criteria. **Table S3.** CASP critical appraisal checklist for analytical qualitative studies. **Table S4.** CASP critical appraisal checklist for analytical qualitative studies.

## Data Availability

All data related to this study are reported in this document.

## Abbreviations

UNICEF: United Nations Children's Fund
GCM/CM: Girl Child Marriage/ Child Marriage
EM: Early Marriage
STD: Sexually Transmitted Diseases
SEM: Socio-Ecological Mode
CDC: Centers for Disease Control and Prevention
CASP: Critical Appraisal Skills Program
Mesh: Medical Subject Headings
PRISMA: Preferred Reporting Items for Systematic Reviews and Meta-Analyses
ENTREQ: Enhancing Transparency in the Qualitative Research Synthesis Report
