# Supplementary material for: Socio-ecological factors of girl child marriage: a meta-synthesis of qualitative research
Source: BMC Public Health. 2024 Feb 10;24:428. doi: 10.1186/s12889-023-17626-z (PMC10858465; doi:10.1186/s12889-023-17626-z)
Supplement: Supplementary file 1 — Additional file 1: Table S1. Enhancing transparency in reporting the synthesis of qualitative research: the ENTREQ statement. Table S2. Inclusion and exclusion criteria. Table S3. CASP critical appraisal checklist for analytical qualitative studies. Table S4. CASP critical appraisal checklist for analytical qualitative studies. [file 12889_2023_17626_MOESM1_ESM.docx]

**Table S1.** Enhancing transparency in reporting the synthesis of qualitative research: the ENTREQ statement

| No | Item | Guide and description | Page number |
| --- | --- | --- | --- |
| 1 | Aim | State the research question the synthesis addresses. | 4 |
| 2 | Synthesis methodology | Identify the synthesis methodology or theoretical framework which underpins the synthesis, and describe the rationale for choice of methodology (e.g. meta-ethnography, thematic synthesis, critical interpretive synthesis, grounded theory synthesis, realist synthesis, meta-aggregation, meta-study, framework synthesis). | 5-6 |
| 3 | Approach to searching | Indicate whether the search was pre-planned (comprehensive search strategies to seek all available studies) or iterative (to seek all available concepts until they theoretical saturation is achieved). | 4 |
| 4 | Inclusion criteria | Specify the inclusion/exclusion criteria (e.g. in terms of population, language, year limits, type of publication, study type). | 5 |
| 5 | Data sources | Describe the information sources used (e.g. electronic databases (MEDLINE, EMBASE, CINAHL, psycINFO, Econlit), grey literature databases (digital thesis, policy reports), relevant organisational websites, experts, information specialists, generic web searches (Google Scholar) hand searching, reference lists) and when the searches conducted; provide the rationale for using the data sources. | 4 |
| 6 | Electronic Search strategy | Describe the literature search (e.g. provide electronic search strategies with population terms, clinical or health topic terms, experiential or social phenomena related terms, filters for qualitative research, and search limits). | 4 |
| 7 | Study screening methods | Describe the process of study screening and sifting (e.g. title, abstract and full text review, number of independent reviewers who screened studies) | Figure2 |
| 8 | Study characteristics | Present the characteristics of the included studies (e.g. year of publication, country, population, number of participants, data collection, methodology, analysis, research questions). | 6 |
| 9 | Study selection results | Identify the number of studies screened and provide reasons for study exclusion (e,g, for comprehensive searching, provide numbers of studies screened and reasons for exclusion indicated in a figure/flowchart; for iterative searching describe reasons for study exclusion and inclusion based on modifications t the research question and/or contribution to theory development). | Figure2 |
| 10 | Rationale for appraisal | Describe the rationale and approach used to appraise the included studies or selected findings (e.g. assessment of conduct (validity and robustness), assessment of reporting (transparency), assessment of content and utility of the findings). | 6 |
| 11 | Appraisal items | State the tools, frameworks and criteria used to appraise the studies or selected findings (e.g. Existing tools: CASP, QARI, COREQ, Mays and Pope [25]; reviewer developed tools; describe the domains assessed: research team, study design, data analysis and interpretations, reporting). | 6 |
| 12 | Appraisal process | Indicate whether the appraisal was conducted independently by more than one reviewer and if consensus was required. | 5-6 |
| 13 | Appraisal results | Present results of the quality assessment and indicate which articles, if any, were weighted/excluded based on the assessment and give the rationale. | 6 |
| 14 | Data extraction | Indicate which sections of the primary studies were analyzed and how were the data extracted from the primary studies? (e.g. all text under the headings “results /conclusions” were extracted electronically and entered into a computer software). | 5 |
| 15 | Software | State the computer software used, if any | 6 |
| 16 | Number of reviewers | Identify who was involved in coding and analysis. | 5 |
| 17 | Coding | Describe the process for coding of data (e.g. line by line coding to search for concepts) | 5-6  Figure1 |
| 18 | Study comparison | Describe how were comparisons made within and across studies (e.g. subsequent studies were coded into pre-existing concepts, and new concepts were created when deemed necessary). | 5-6  Figure1 |
| 19 | Derivation of themes | Explain whether the process of deriving the themes or constructs was inductive or deductive. | 5-6  Figure1 |
| 20 | Quotations | Provide quotations from the primary studies to illustrate themes/constructs, and identify whether the quotations were participant quotations of the author’s interpretation. | Table2 |
| 21 | Synthesis output | Present rich, compelling and useful results that go beyond a summary of the primary studies (e.g. new interpretation, models of evidence, conceptual models, analytical framework, development of a new theory or construct) | 7-12 |

**Table S2.** Inclusion and exclusion criteria

| **Exclusion Criteria** | **Inclusion Criteria** |  |
| --- | --- | --- |
| women who got married at the age of 18 and higher.  Boys who got married early | Population get(women) married under 18 years | Participants |
| Controlled trials, Quantitative designs, quasi experimental studies, and pre-test/post-test studies, reports, letter to editor, mixed method, systematic review | All of Qualitative study (grounded theory, phenomenology, ethnography, content analysis) | Study Type |
| - | No intervention | Intervention |
| - | Any type of socio-health setting | Setting |
| - | Driver of child marriage | Outcomes |
| Scientific articles before 2000 | Scientific full text articles published in indexed scientific journals | Dissemination Type |
| Articles written in languages other than English | English | Language |
| Lack of access to the full text | Access to the full text of the article | Others |

**Table S3**. CASP critical appraisal checklist for analytical qualitative studies

|  | Critical appraisal checklist | Yes  3 | No  1 | Can’t Tell  2 |
| --- | --- | --- | --- | --- |
| 1 | Was there a clear statement of the aims of the research? | 34 | 0 | 0 |
| 2 | Is a qualitative methodology appropriate? | 34 | 0 | 0 |
| 3 | Was the research design appropriate to address the aims of the research? | 33 | 0 | 1 |
| 4 | Was the recruitment strategy appropriate to the aims of the research? | 31 | 0 | 3 |
| 5 | Was the data collected in a way that addressed the research issue? | 29 | 0 | 5 |
| 6 | Has the relationship between researcher and participants been adequately considered? | 8 | 24 | 2 |
| 7 | Have ethical issues been taken into consideration? | 27 | 6 | 1 |
| 8 | Was the data analysis sufficiently rigorous? | 23 | 8 | 3 |
| 9 | Is there a clear statement of findings? | 32 | 1 | 1 |
| 10 | How valuable is the research? | 31 | 0 | 3 |

**Table S4.** CASP critical appraisal checklist for analytical qualitative studies

|  | **1** | **2** | **3** | **4** | **5** | **6** | **7** | **8** | **9** | **10** | **Total**  **Score (24)** |
| --- | --- | --- | --- | --- | --- | --- | --- | --- | --- | --- | --- |
| Chowdhury | Y | Y | Y | Can Not | Can Not | N | Y | N | Y | Y | 18 |
| James | Y | Y | Y | Y | Y | N | Y | N | Y | Y | 20 |
| Matlabi | Y | Y | Can Not | Y | Y | N | N | N | Y | Can Not | 16 |
| Sabbe | Y | Y | Y | Y | Y | N | Y | Can Not | Y | Y | 21 |
| Nasrullah | Y | Y | Y | Y | Y | N | Y | Can Not | Can Not | Can Not | 19 |
| Vang | Y | Y | Y | Y | Y | N | Can Not | Y | Y | Y | 21 |
| Montazeri | Y | Y | Y | Y | Y | N | Y | Y | Y | Y | 22 |
| Segal | Y | Y | Y | Y | Y | N | Y | Y | Y | Y | 22 |
| Syamsidah, | Y | Y | Y | Y | Y | N | N | N | N | Can Not | 15 |
| Mangeli | Y | Y | Y | Y | Y | N | Y | Y | Y | Y | 22 |
| Mourtada | Y | Y | Y | Y | Y | N | Y | Y | Y | Y | 22 |
| Iustitiani | Y | Y | Y | Can Not | Can Not | N | Y | N | Y | Y | 18 |
| Mcdougal | Y | Y | Y | Y | Y | N | Y | Y | Y | Y | 22 |
| Muhith | Y | Y | Y | Can Not | Can Not | N | N | N | Y | Y | 16 |
| Stark | Y | Y | Y | Y | Y | Y | Y | Y | Y | Y | 24 |
| Aleksandrova | Y | Y | Y | Y | Y | Y | Y | Y | Y | Y | 24 |
| Bhandari | Y | Y | Y | Y | Can Not | N | N | N | Y | Y | 17 |
| Cislaghi | Y | Y | Y | Y | Y | Y | Y | Y | Y | Y | 24 |
| Dean | Y | Y | Y | Y | Y | N | Y | Y | Y | Y | 22 |
| Judy | Y | Y | Y | Y | N | N | N | Can Not | Y | Y | 17 |
| Kohno | Y | Y | Y | Y | Y | Y | Y | Y | Y | Y | 24 |
| Lebni | Y | Y | Y | Y | Y | N | Y | Y | Y | Y | 22 |
| Kohno | Y | Y | Y | Y | Y | Y | Y | Y | Y | Y | 24 |
| Madut | Y | Y | Y | Y | Can Not | Y | Y | Y | Y | Y | 23 |
| Elnakib | Y | Y | Y | Y | Y | Y | Y | Y | Y | Y | 24 |
| Mirzaee | Y | Y | Y | Y | Y | Can Not | Y | Y | Y | Y | 23 |
| Mrayan | Y | Y | Y | Y | Y | N | Y | Y | Y | Y | 22 |
| Neema | Y | Y | Y | Y | Y | N | Y | Y | Y | Y | 22 |
| Schaffniti | Y | Y | Y | Y | Y | Can Not | Y | Y | Y | Y | 23 |
| Susilo | Y | Y | Y | Y | Y | N | Y | N | Y | Y | 20 |
| Baraka | Y | Y | Y | Y | Y | N | Y | Y | Y | Y | 22 |
| Maghsoudi | Y | Y | Y | Y | Y | N | N | Y | Y | Y | 20 |
| Bozorgi | Y | Y | Y | Y | Y | Can Not | Y | Y | Y | Y | 23 |
| Tewahido | Y | Y | Y | Y | Y | N | Y | Y | Y | Y | 22 |
